# Rationing of Nursing Care and Professional Burnout Among Nurses Working in Cardiovascular Settings

**DOI:** 10.3389/fpsyg.2021.726318

**Published:** 2021-10-06

**Authors:** Izabella Uchmanowicz, Grzegorz Kubielas, Bogusława Serzysko, Anna Kołcz, Piotr Gurowiec, Ewelina Kolarczyk

**Affiliations:** ^1^Department of Clinical Nursing, Faculty of Health Sciences, Wrocław Medical University, Wrocław, Poland; ^2^Department of Healthcare, Higher School of Applied Sciences in Ruda Śląska, Ruda Śląska, Poland; ^3^Laboratory of Ergonomics and Biomedical Monitoring, Wrocław Medical University, Wrocław, Poland; ^4^Faculty of Health Sciences, University of Opole, Opole, Poland; ^5^Department of Gerontology and Geriatric Nursing, Faculty of Health Sciences, Medical University of Silesia, Katowice, Poland

**Keywords:** rationing of nursing care, burnout, nurses, BERNCA-R, MBI, job satisfaction, emotional exhaustion, depersonalisation

## Abstract

**Introduction:** Nursing needs close interpersonal contact with the patient and emotional involvement, therefore can contribute to professional burnout and rationing of nursing care.

**Aim:** Assessing the relationship between the rationing of nursing care and professional burnout in nursing staff.

**Materials and Methods:** The study included a group of 219 nurses working in cardiovascular facilities. This was a cross-sectional study designed to investigate the relationship between factors of the care rationing and professional burnout. The survey data was collected with standardised and research instruments such as the revised Basel Extent of Rationing of Nursing Care questionnaire (BERNCA-R) and the Maslach Burnout Inventory (MBI).

**Results:** The total mean BERNCA-R score was 1.38 (SD = 0.62), while the total MBI score amounted to 38.14 (SD = 22.93). The specific components of professional burnout yielded the values: emotional exhaustion (*M* = 44.8), job dissatisfaction (*M* = 40.66), and depersonalisation (*M* = 28.95). Multiple linear regression showed that independent predictors of BERNCA-R score were emotional exhaustion, depersonalisation, job dissatisfaction, and multi-jobs activity (*p* < 0.001).

**Conclusion:** The level of rationing of nursing care in cardiovascular facilities increases along with emotional exhaustion, depersonalisation and job dissatisfaction, and multi-jobs activity.

## Introduction

Rationing of nursing care is defined as a complete or partial omission of the required patient care. This phenomenon has been first described by Kalisch (2006), an American nurse. Kalisch (2006) have proposed a conceptual model of missed nursing care. According to this model, the rationing of nursing care results from several malfunction factors: insufficient resources to provide patients with required care (reduction in nurse employment), time-consuming handling on new technologies used in medical care, medical record-keeping, individual factors (tiredness, family issues), working environment factors (conflicts among co-workers), medical facility management system (numerous changes to the organisation and senior management), and communication (Kalisch et al., 2009; Wakefield, 2014).

Given priorities of nursing tasks and malfunctioning individual and organisational factors, the nurses are forced to selectively perform care tasks, and specific nursing tasks may be omitted or delayed (Ulrich, 2016). In addition, care rationing could also be affected by the nurses’ clinical knowledge, attitudes, and judgements toward allocating resources for the patients (Schubert et al., 2008). The nursing profession depends on the skills and acquired competencies of nurses, but also specific personality traits and optimistic attitudes about the world and people. According to recent reports, the more optimistic thinking and higher job satisfaction, the lower risk of missed nursing care (Jaworski et al., 2020; Uchmanowicz et al., 2020).

Rationing of nursing care has been reported worldwide. It poses a direct threat to patient safety (Kalisch et al., 2009; Uchmanowicz and Gotlib, 2018). Prior research suggests that this issue applies to nearly 84% of nurses, who miss at least 1 out of 15 nursing tasks (Hessels et al., 2015; Park et al., 2018). As reported by Liu et al. (2018), rationing of nursing care and nurse burnout were mediators negatively associated with patient safety. Rationing of nursing care is one of the leading causes underlying adverse events and medical errors, posing a threat to patient’s health and lives (Hessels et al., 2019). In the study by O’Connor et al. (2017), medical errors were associated with professional burnout in approximately 64% of cases.

The findings reviewed here implicate the significant relationship between the rationing of nursing job satisfaction and the risk of professional burnout (Ntantana et al., 2017). For example, the existing studies demonstrate the strong correlation between nurses’ job satisfaction and patients’ satisfaction with healthcare services (Liu et al., 2016). It was shown that lower job satisfaction increased missed nursing care (Blackman et al., 2015). Fewer professional success and more failures reported by nurses were due to close interpersonal contact with the patient and excessive involvement in nursing care (Rizo-Baeza et al., 2018).

Professional burnout is associated with poor psychological wellbeing related to emotional exhaustion, negative assessment of personal and depersonalisation (Hall et al., 2016). Zander et al. (2014) reported the associations between inefficient leadership, inadequate organisation of nursing work, as well as high emotional exhaustion and rationing of nursing care. These reports suggest that the relationship between burnout, missed nursing care, and individual factors should be examined more closely. According to Maslach et al. (2001), emotional exhaustion is manifested by discouragement toward work, lowered and pessimistic professional activity, psycho-physical tension (irritability) and somatic conditions, including tiredness, headaches, insomnia, gastric disorders, and susceptibility to regular colds. Reduced personal accomplishment is characterised by low confidence in one’s own capabilities, no satisfaction with professional achievements, inability to solve professional issues, and an aggressive and avoidant attitude in relations with others. Depersonalisation is described by indifference, cynicism, superficiality, shortening contact time, and distancing from the patients’ care needs. Healthcare personnel belong to the professional group at particularly high risk of burnout phenomenon due to specific organisational conditions, individual characteristics of the nursing profession and nursing requirements. This condition results from failure to adapt to the working environment and it negatively impacts work effectiveness and social and family life (Maslach et al., 2001). Professional burnout became a serious social issue, diminishing the quality of provided nursing care (Bridgeman et al., 2018). Several studies have found that work-related stress nursing is associated with burnout, job satisfaction, and diminished physical and mental health outcomes (Khamisa et al., 2015). Stressors contributing to work-related stress and increased professional burnout include experiences of conflicts with peers and patients, high job demands, and overtime (Garrosa et al., 2010; Nowrouzi et al., 2015). The recent research also shows the effects of professional burnout among medical personnel on patient dissatisfaction with provided medical care. Diminished satisfaction with work, professional burnout and frustration caused by negligible effects of provided care on the patient health are found primarily among the bedside nurses working directly with patients in hospitals or at homes. Overall, lower patient satisfaction with the quality of care is observed in healthcare facilities, where nurses often experience burnout and dissatisfaction with their working conditions (McHugh et al., 2011). The literature shows that there is the association between healthcare professionals’ well-being and burnout with regard patient safety.

To date, the literature attempted to determine the associations between professional burnout and rationing of nursing care (Kalisch et al., 2011). However, some important issues have been omitted because the prior research was more focused on nurses’ job satisfaction. It seems that studies analysing specific determinants linking job dissatisfaction, professional burnout, and exhaustion are insufficient. There is still a gap in research investigating professional burnout given specific healthcare organisations and cultural settings in the countries. The findings of professional burnout and the missed nursing care cannot be easily generalised because the existing studies cover various healthcare facilities employing nurses and consider highly specific medical environments. There is little evidence of the above-mentioned adverse phenomena among nurses working in cardiology departments. Therefore, the aim of this study was to assess the relationship between the rationing of nursing care and professional burnout in nursing staff.

## Materials and Methods

### Study Group Selection

The study used a cross-sectional design and analysed the diagnostic survey data selected collected from a group of nurses working at non-invasive cardiology wards of four hospitals in Wrocław, Poland. It was carried out between January and May 2020. The study included 219 nurses based on the following inclusion criteria: (1) consent to participate in the survey, (2) valid nursing licence, (3) working full time, and (4) no recent sick leave or holiday leave exceeding 3 months.

### Study Procedure

As the study was based on the cross-sectional design, the Strengthening the Reporting of Observational Studies in Epidemiology (STROBE) guidelines were followed (von Elm et al., 2007). The research was performed upon approval of the Bioethics Committee of the Wrocław Medical University, Poland. The subjects were informed that their participation is voluntary and anonymous. Upon obtaining written consent, the surveys were conducted in line with the recommendations of Good Clinical Practice (Vijayananthan and Nawawi, 2008) and the Helsinki Declaration of the World Medical Association (2013).

### Description of the Questionnaire and the Applied Measures

The Basel Extent of Rationing of Nursing Care questionnaire (BERNCA-R) questionnaire was elaborated by Schubert et al. (2007). This instrument has proven to be effective for evaluating the rationing of nursing care in a hospital environment. The study used the Polish language version of the questionnaire adapted by Uchmanowicz et al. (2019). The surveys data were collected using the standardised research instruments: the revised BERNCA-R and the Maslach Burnout Inventory (MBI).

The BERNCA-R evaluates the frequency of rationing of nursing care by the respondents. It consists of 32 items, examining events and situations in which care rationing happens to occur. Each question is scored according to the 4-point scale, where 0 = “There was no need”; 1 = “Never”; 2 = “Rarely”; 3 = “Sometimes”; 4 = “Often.” The total score of the care rationing is the mean across 32 questions. The total care rationing score ranges from 0 to 4 and can be interpreted analogically to the interpretation of a single item (Uchmanowicz et al., 2019).

Burnout was measured using the MBI, a validated, standardised Polish adaptation by Pasikowski (2006). The MBI assesses a professional burnout level on three subscales: emotional exhaustion, depersonalisation, and lack of job satisfaction. Each subscale score ranges from 0 to 100 points, where the higher score indicates higher professional burnout. In addition, the total burnout rate is the mean score of the three subscales. There are no standards for evaluating the levels of respondents’ professional burnout. The assessments of professional burnout in nurses based on MBI have been reported worldwide and proven effective for the measures of burnout reduction in healthcare policy planning (Poghosyan et al., 2009; Choi et al., 2019).

In addition, the study used a self-designed socio-demographic survey including the questions about age, job experience, education, specialisations, multi-jobs activity at healthcare centres, number of patients, and marital status.

### Statistical Methods

The survey data were systematised and arranged by quantitative and qualitative variables. The analysis of quantitative variables included the mean (*M*), standard deviation (SD), median, quartiles, minimum, and maximum. For qualitative variables, the frequency and percentage of occurrences were calculated. The group differences in the BERNCA-R scores were examined with the Mann–Whitney test. Comparisons between three or more groups was performed with the Kruskal–Wallis test. Identification of statistically significant effects was performed with the *post hoc* Dunn test. Correlations between were analysed using Spearman’s correlation coefficient. Multiple regression analysis was used to examine the impact of explanatory variables on the BERNCA-R score. The resulting quality of the model was assessed by the *R*^2^ determination coefficient. The regression model parameters included the 95% confidence intervals. The results were considered statistically significant at the level of *p* < 0.05. Calculations were performed using the R Core Team, 4.0.2 version (R Core Team, 2019).

## Results

### Study Group

The study included 219 cardiac nurses. The majority of the respondents (81.28%) declared living with a partner. The largest number of respondents reported bachelor (36.99%) or secondary education (33.79%), while higher education (master’s degree) was indicated by 18.26% of respondents. Only 10.96% of respondents reported other medical education. And 17.81% of respondents completed postgraduate studies with a specialisation in nursing. The most numerous group included nurses with 20-years of working experience (55.25%), while 21.92% reported the shortest working experience. Approximately 5% of respondents had 10- or 15-year job experience. The highest number of respondents declared one job (68.49%); two jobs were declared by 25.11% of respondents, while 14 persons (6.39%) reported more than two jobs. The respondents usually declared that a salary level matters (91.78%), while nine persons had no opinion on this subject (4.11%), and only four respondents (1.83%) believed that salary is insignificant for job satisfaction. Nearly half of the respondents (43.84%) reported nursing care to 16–25 patients, and 31.05% of respondents declared up to 16 patients, approximately 9% of respondents provided care to 26–35 patients; 36 of respondents declared providing care to more than 35 patients (16.44%). The detailed sociodemographic characteristics are shown in Table 1.

**TABLE 1:** Characteristics of study participants (*n* = 219).

Parameter		*n*	%
Age	23–30 years	41	18.72
	31–40 years	29	13.24
	41–50 years	89	40.64
	>50 years	60	27.40
Job seniority	0–5 years	48	21.92
	6–10 years	11	5.02
	11–15 years	13	5.94
	16–20 years	26	11.87
	>20 years	121	55.25
Education	MSc in nursing/obstetrics	40	18.26
	Bachelor’s degree in nursing/obstetric	81	36.99
	Medical secondary school	74	33.79
	Medical college and “other” education	24	10.96
Specialisation	No	180	82.19
	Yes	39	17.81
Number of working places?	One	150	68.49
	Two	55	25.11
	Three or four	14	6.39
Patient-to-nurse ratio	1–15 patients	68	31.05
	16–25 patients	96	43.84
	26–35 patients	19	8.68
	>35 patients	36	16.44
Marital status	In a relationship	178	81.28
	Single	41	18.72

### Rationing of Nursing Care

The analysis demonstrated that the mean total BERNCA-R score was 1.38 (SD = 0.62). The highest results for rationed nursing tasks were observed for activating and rehabilitating care (*M* = 2.03), studying the situation of individual patients and care plans at the beginning of shift (*M* = 1.8), assessment of newly admitted patients’ needs (*M* = 1.84), providing emotional or psycho-social support to the patient (*M* = 1.79) as well as administration of prescribed medication or infusion on time (*M* = 1.77).

According to the Mann–Whitney test, the frequency of rationing of nursing care was higher among nurses aged 41–50 and >50 compared to those aged 23–30 and 31–40 (*p* = 0.001). In addition, rationing of nursing care was higher among respondents with 6–10 years of job experience compared to those with 16–20 and >20 years of job experience (*p* = 0.001). Moreover, a higher level of care rationing was observed in respondents with 0–5 years of job experience than nurses with more than 20 years of working experience (*p* = 0.001). The Kruskal–Wallis statistics demonstrated increased nursing care rationing in nurses with a bachelor’s degree compared to other educational levels (*p* = 0.001). Detailed results are presented in Table 2.

**TABLE 2:** Demographic characteristics of the study group compared to the rationing of nursing care.

Parameter	Group	BERNCA-R (score)			*p*
		Mean ± SD	Median	Quartiles	
Age	23–30 years (*N* = 41) – A	1.57 ± 0.67	1.59	1.47–1.78	*p* = 0.001*
	31–40 years (*N* = 29) – B	1.61 ± 0.44	1.56	1.34–1.84	
	41–50 years (*N* = 89) – C	1.32 ± 0.61	1.47	1–1.69	B.A > C.D
	>50 years (*N* = 60) – D	1.22 ± 0.65	1.4	1.03–1.53	
Job seniority	0–5 years (*N* = 48) – A	1.58 ± 0.64	1.58	1.34–1.84	*p* = 0.001*
	6–10 years (*N* = 11) – B	1.73 ± 0.23	1.75	1.53–1.81	
	11–15 years (*N* = 13) – C	1.41 ± 0.59	1.66	1.25–1.69	B > D.E A > E
	16–20 years (*N* = 26) – D	1.36 ± 0.81	1.53	0.86–1.76	
	>20 years (*N* = 121) – E	1.26 ± 0.57	1.44	1.16–1.59	
Education	MSc in nursing/obstetrics (*N* = 40) – A	1.24 ± 0.79	1.39	0.74–1.78	*p* = 0.01*
	Bachelor’s degree in nursing/obstetrics (*N* = 81) – B	1.55 ± 0.6	1.56	1.34–1.78	
	Medical secondary school (*N* = 74) – C	1.32 ± 0.52	1.46	1.25–1.56	B > C.A.D
	Medical college and “other” education (*N* = 24) – D	1.21 ± 0.59	1.48	0.86–1.61	
Specialisation	No (*N* = 180)	1.42 ± 0.61	1.48	1.25–1.67	*p* = 0.476
	Yes (*N* = 39)	1.2 ± 0.67	1.5	0.72–1.77	
Number of working places	One (*N* = 150)	1.43 ± 0.58	1.53	1.34–1.71	*p* = 0.089
	Two (*N* = 55)	1.21 ± 0.64	1.44	0.84–1.59	
	Three or four (*N* = 14)	1.46 ± 0.95	1.31	0.76–1.78	
Patient-to-nurse ratio	1–15 patients (*N* = 68)	1.31 ± 0.63	1.47	1.04–1.72	*p* = 0.64
	16–25 patients (*N* = 96)	1.43 ± 0.55	1.5	1.34–1.69	
	26–35 patients (*N* = 19)	1.32 ± 0.97	1.47	0.53–2.08	
	>35 patients (*N* = 36)	1.4 ± 0.59	1.46	1.18–1.57	
Marital status	In a relationship (*N* = 178)	1.35 ± 0.62	1.47	1.17–1.66	*p* = 0.084
	Single (*N* = 41)	1.5 ± 0.64	1.53	1.28–1.78	

*p* – comparison of two groups: Mann–Whitney test; comparison of >two groups: Kruskal–Wallis test + *post hoc* analysis (Dunn test). *Statistically significant correlation (*p* < 0.05).

### Professional Burnout

The MBI measures demonstrated that the mean burnout rate across all subscales was 38.14 (SD = 22.93). The main components of professional burnout in respondents were emotional exhaustion (*M* = 44.8), followed by job dissatisfaction (*M* = 40.66) and depersonalisation (*M* = 28.95), respectively.

Analysis of Spearman’s correlation coefficient showed the positive correlations between the BERNCA-R score and the total MBI score (*r* = 0.249, *p* < 0.001) and the subscales of emotional exhaustion (*r* = 0.287, *p* < 0.001) and depersonalisation (*r* = 0.383, *p* < 0.001). The results are presented in Table 3.

**TABLE 3:** Correlations between professional burnout and rationing of nursing care.

MBI	BERNCA-R
	Spearman’s correlation coefficient
Overall MBI score	*r* = 0.249*
Emotional exhaustion	*r* = 0.287*
Depersonalisation	*r* = 0.383*
Lack of job satisfaction	*r* = −0.042

*Statistically significant correlation (*p* < 0.05).

### Basel Extent of Rationing of Nursing Care questionnaire Independent Predictor

For the regression model combining the burnout subscales, the *R*^2^ determination coefficient was 36.04%. The regression analysis demonstrated that the independent predictors of BERNCA-R score were emotional exhaustion, depersonalisation, lack of job satisfaction (all *p* < 0.001); the analysis showed that the significant predictor was also job activity at three or four healthcare centres (*p* = 0.038). The regression results of the model for the subscales combined are presented in Table 4.

**TABLE 4:** Multiple regression model of nursing care rationing for the MBI subscales.

Characteristic		Parameter	95% CI		*p*
Age	23–30 years	ref.			
	31–40 years	0.019	–0.326	0.365	0.912
	41–50 years	–0.091	–0.468	0.285	0.634
	>50 years	–0.016	–0.439	0.407	0.942
Job experience	0–5 years	ref.			
	6–10 years	0.084	–0.343	0.511	0.7
	11–15 years	–0.051	–0.492	0.39	0.82
	16–20 years	–0.075	–0.469	0.32	0.711
	>20 years	–0.289	–0.67	0.092	0.139
Education	MSc in nursing/obstetrics	ref.			
	Bachelor’s degree in nursing/obstetrics	0.072	–0.144	0.288	0.514
	Medical secondary school	–0.006	–0.252	0.24	0.96
	Medical college and “other” education	–0.247	–0.534	0.041	0.094
Specialisation	No	ref.			
	Yes	–0.024	–0.221	0.174	0.815
Number of working places	One	ref.			
	Two	–0.057	–0.231	0.117	0.523
	Three or four	0.335	0.02	0.65	0.038*
Patient-to-nurse ratio	1–15 patients	ref.			
	16–25 patients	0.082	–0.096	0.259	0.369
	26–35 patients	–0.042	–0.321	0.237	0.767
	>35 patients	–0.023	–0.251	0.206	0.846
Marital status	In a relationship	ref.			
	Single	0.085	–0.104	0.274	0.377
Emotional exhaustion		0.008	0.005	0.012	< 0.001*
Depersonalisation		0.006	0.003	0.009	< 0.001*
Lack of job satisfaction		–0.007	–0.01	–0.003	< 0.001*

*p* – multiple linear regression. *Statistical significance for *p* < 0.05.

For the regression model testing the explanatory variable of the total MBI score, the *R*^2^ determination coefficient was 23.31% (see Table 5). The model indicated that overall burnout score was the predictor of the rationing of nursing care (*p* < 0.001).

**TABLE 5:** Multiple regression model of nursing care rationing for the overall MBI score.

Feature		Parameter	95% CI		*p*
Age	23–30 years	ref.			
	31–40 years	0.046	–0.323	0.415	0.806
	41–50 years	–0.093	–0.498	0.311	0.652
	>50 years	–0.068	–0.516	0.381	0.768
Work experience	0–5 years	ref.			
	6–10 years	0.116	–0.344	0.576	0.622
	11–15 years	–0.089	–0.568	0.39	0.716
	16–20 years	–0.132	–0.557	0.294	0.545
	>20 years	–0.264	–0.671	0.142	0.204
Education	Master of nursing/midwifery	ref.			
	Bachelor of nursing/midwifery	0.177	–0.055	0.409	0.136
	Medical school	–0.027	–0.294	0.24	0.843
	Medical college and “other” education	–0.105	–0.414	0.204	0.505
Specialisation	No	ref.			
	Yes	–0.029	–0.244	0.185	0.79
Number of jobs	One	ref.			
	Two	–0.111	–0.3	0.077	0.249
	Three or four	0.16	–0.176	0.496	0.351
Number of patients cared for	1–15 patients	ref.			
	16–25 patients	0.066	–0.127	0.259	0.504
	26–35 patients	–0.156	–0.458	0.145	0.312
	>35 patients	0.034	–0.213	0.281	0.787
Civil status	In the relationship	ref.			
	Single	0.115	–0.089	0.32	0.271
Overall MBI score		0.01	0.006	0.014	< 0.001*

*p* – multiple linear regression. *Statistical significance for *p* < 0.05.

## Discussion

This study examined the relationship between rationing of nursing care and professional burnout in nurses working in cardiovascular settings. The study showed that rationing of nursing care correlated positively with total MBI score and subscales of emotional exhaustion and depersonalisation. In the study by Rizo-Baeza et al. (2018), the factors affecting professional burnout of nurses working at palliative care wards included extensive work (more than 6 h a day), medium and high workload, raising a child alone, the unfriendly working condition (decreased personal accomplishments). The above study showed that emotional exhaustion occurred in 37.3% of respondents, while depersonalisation in the percentage of 35.1%. Our analyses of the nurse sample found that professional burnout was primarily underlaid by emotional exhaustion, while depersonalisation was less frequent.

Nurses belong to the professional group with the highest risk of professional burnout because of continuous exposure to stressful situations at work. The main reason behind this unfavourable phenomenon is the psycho-social nature of contacts with other humans seeking care and help, which requires a continuous nurses’ focus on the other person’s needs. The study by Khamisa et al. (2013) revealed that stress at work resulting from relations with other personnel affects each aspect of professional burnout. Shortage of nursing staff causes significant emotional exhaustion (16%), depersonalisation (13%), and negative assessment of personal accomplishments (10%). We obtained similar findings because respondents’ professional burnout in respondents arose mainly from emotional exhaustion (*M* = 44.8) and depersonalisation (*M* = 28.95), as indicated by the positive correlations. In literature, the surveyed nurses, mostly aged 51 and above (51%), 62% had more than 1 year of job experience, while 62% had a higher education degree. Similar results were demonstrated by our research, showing that higher education was held by the majority of respondents. At the same time, rationing of nursing care occurred most frequently in the respondents with the bachelor’s degree. This finding suggests the effect of the young age of nurses who land their first job and have no professional experience. Similarly to our research, the vast majority of respondents was aged 41–50 and above, while the results showed that rationing of nursing care occurred most frequently among nurses with secondary education. In addition, the present analysis demonstrated that emotional exhaustion, depersonalisation, job dissatisfaction, and multi-jobs activity (at more than two centres) were significantly correlated with rationing of nursing care (*p* < 0.001). Given the data available in this study, one cannot determine the casual pathway between rationing of nursing care, nurse burnout, and stress. This study aimed at investigating rationing of nursing care, but not analysing their stress levels. Examinations of stress in nursing staff should be considered in future studies.

The present study analysed the associations between the socio-demographic characteristics of a sample of cardiovascular nurses and the rationing of nursing care. Following the other researchers’ approach, increased susceptibility to stress and emotional exhaustion are associated with a subjective feeling of getting tired of work. Such tiredness decreases the capacity to work and motivation to continue it, and increases the risk of making medical errors (Chang et al., 2009). The study by Młynarska et al. (2020) demonstrated that the greater tiredness, the more frequent rationing of nursing care at emergency wards. Similar findings were reported by research on the effects of the socio-demographic factors on missed nursing care. For example, according to Młynarska et al. (2020), gender, age, residence, education, job experience, and type of employment regarding to working hours have no impact on the rationing of nursing care. In contrast to our study, suggesting the negative impact of the number of hours on rationing of nursing care (*p* = 0.038).

One recognises the feeling of lack of job satisfaction as a critical cause of professional burnout syndrome. It stems from many unfavourable phenomena and situations specific to the nursing profession. In this study, rationing of nursing care was higher in nurses reporting a lack of job satisfaction (*p* < 0.001). Zeleníková et al. (2020) found that the relationship between job satisfaction and rationing of nursing care depended on nurses’ working environment and interpersonal relations among medical personnel. These results were confirmed by an international study by Bragadóttir et al. (2020) performed on 7.079 nurses from Turkey, Australia, United States and Ireland, aged up to 45. The authors demonstrated that job satisfaction was associated with rationing of nursing care and influenced by the country, the nursing team position, type of ward and hospital, professional experience, and human resources. These findings are consistent with our study, suggesting that job dissatisfaction affects the rationing of nursing care. In contrast to the above-mentioned findings, the present study suggests that the age of nursing staff can affect the rationing of nursing care. It occurs more frequently in older nurses and those with shorter job experience. These differences can be explained by the younger age of respondents studied by Bragadóttir et al. (2020) and usage of other diagnostic measurements. A multicentre study by Jaworski et al. (2020) obtained similar results where older nurses and nurses with the shortest job experience rationed nursing care more often. According to the authors, low job satisfaction and pessimistic attitude to reality increase the rationing of nursing care (Jaworski et al., 2020).

To date, there have been reports on the relationship between nurses’ job satisfaction and remuneration and overtime hours (Khamisa et al., 2013). In the study by Serafin et al. (2019), the nurses working at Swedish surgical wards had higher job satisfaction than their Polish counterparts. These results for the Polish nurses can be explained by dissatisfaction with their remuneration, which is in line with our study and other reports above referred. The issue of rationing of nursing care in Sweden was surveyed by Ball et al. (2016), who revealed that this phenomenon applies to nearly 74% of nurses and may be associated with the working environment. In our study, the remuneration amount had no impact on the rationing of nursing care compared to the results concerning professional burnout. In Sweden, rationing was associated primarily with working shifts, human resources, and the number of patients under nursing care. According to Maslach et al. (2001), improvements in patient satisfaction and better protection against undesired effects due to improper nursing care depend on improving job satisfaction of nurses. A U*nited* S*tates* study concerning 95.499 nurses demonstrated that lack of job satisfaction and professional burnout were associated primarily with nurses’ dissatisfaction with provided healthcare services (McHugh et al., 2011).

Our study indicated that the component of depersonalisation burnout affected rationing of nursing care, served mainly by older nurses (41–50 and >50). Other research on professional burnout among nurses have reported similar correlations. For example, according to Dębska et al. (2019), Polish nurses were significantly older than Slovakian and Czech nurses, and they, importantly, exhibited depersonalisation (*p* = 0.055) and negative assessment of personal accomplishment (*p* = 0.058). Similarly, Parola et al. (2017) indicated high rate of professional burnout occurred among palliative care nurses, where emotional exhaustion (19.5%) and depersonalisation (8.2%) were the key components contributing to this high result.

## Conclusion

All aspects of professional burnout in a sample of Polish nurses, i.e., emotional exhaustion, depersonalisation, and lack of job satisfaction, correlated with rationing of nursing care. Nurse’ jobs activity at several healthcare centres may be the important socio-demographic factor affecting rationing of nursing care in cardiovascular wards.

### Methodological Limitations

The present study used the cross-sectional design ad and the small studied group (*n* = 219) selected from one region of Poland (Lower Silesian Voivodeship). This necessitates caution in generalising our results to general populations of nurses worldwide. However, despite these limitations, the significance of our study is supported by similar findings reported in other studies and publications. The present study contributes to a better understanding of the rationing of nursing care and professional burnout among cardiovascular nurses. Certainly, further research is needed to collect more groups of nurses in a multi-centre condition. Replication this study with the larger data set and more robust longitudinal designs will warrant confirmation of these results.

## Data Availability Statement

The raw data supporting the conclusions of this article will be made available by the authors, without undue reservation.

## Ethics Statement

The studies involving human participants were reviewed and approved by Bioethics Committee of the Wrocław Medical University, Poland. The patients/participants provided their written informed consent to participate in this study.

## Author Contributions

IU contributed to study conception and design, acquisition, analysis and interpretation of data, and drafting and critical revision of the manuscript. GK contributed to acquisition, analysis and interpretation of data, and drafting of the manuscript. BS contributed to study conception and design, and drafting of the manuscript. AK and PG contributed to study conception and design, analysis and interpretation of data, and critical revision of the manuscript. EK contributed to study conception and design, acquisition of data, and drafting of the manuscript. All the authors approved the final version of the manuscript.

## Conflict of Interest

The authors declare that the research was conducted in the absence of any commercial or financial relationships that could be construed as a potential conflict of interest.

## Publisher’s Note

All claims expressed in this article are solely those of the authors and do not necessarily represent those of their affiliated organizations, or those of the publisher, the editors and the reviewers. Any product that may be evaluated in this article, or claim that may be made by its manufacturer, is not guaranteed or endorsed by the publisher.
